# Extensive Cerebral Venous Thrombosis as an Isolated Presentation in a COVID-19-Positive Young Adult

**DOI:** 10.7759/cureus.29561

**Published:** 2022-09-25

**Authors:** Farnoosh J Farzin, Chafika Lasfer, Ivyan Kambal

**Affiliations:** 1 Emergency Medicine, Dubai Health Authority, Dubai, ARE; 2 Emergency Medicine, Fakeeh University Hospital, Dubai, ARE

**Keywords:** thrombotic complications of covid-19, d dimer in covid, covid-19 pandemic, venous thromboembolism, covid 19, cerebral venous thrombosis (cvt)

## Abstract

Due to the increase in the number of severe acute respiratory syndrome coronavirus 2 (SARS-CoV-2) cases globally, more medical case reports are being published showing the different complications of coronavirus disease 2019 (COVID-19). One of the important complications is thrombotic events that occur as a sequela of COVID-19. Here we present a case of a previously healthy male patient in his 30s who presented to the emergency unit experiencing headaches, vomiting, and weakness in his left arm. On examination, he was vitally stable, and fully oriented, but noted to have jerky movements of the left arm; therefore, he was sent for a CT brain scan. Shortly after, he developed a generalized tonic-clonic seizure. After stabilizing the patient, CT brain with cerebral venography was done, which revealed extensive thrombosis of the superior sagittal sinus and bilateral superficial cortical veins. The patient's blood test showed a high D-dimer (4.90 ug/ml), and the COVID-19 polymerase chain reaction (PCR) swab test was positive. It is commonly known that COVID-19 infection presents with fever and respiratory symptoms; however, our case illustrates the thrombotic complication of SARS-CoV-2 infection with no pneumonia or respiratory symptoms with a high level of d-dimer.

## Introduction

Cerebral venous thrombosis (CVT) is an uncommon disease with various spectrums of clinical features; however, the most common clinical presentation is a headache [1]. There are numerous predisposing risk factors that lead to the development of CVT, one of them is prior infection [1-3]. Lately, the coronavirus disease 2019 (COVID-19) pandemic had a devastating impact on the population's morbidity and mortality and has been linked to the development of venous thromboembolic diseases [4-11].

Globally, COVID-19 is well known for its manifestation through respiratory symptoms [12]. On the other hand, COVID-19 has also been linked to an increased risk of thrombosis, leading to various conditions including myocardial infarction, stroke, venous thromboembolism, and the microthrombosis of vital organs [13,14]. With the progression of the pandemic, more medical case reports are emerging describing thrombotic complications of COVID-19 [15-17]. Due to the rapidly rising number of COVID-19 infections globally, the severity of CVT as a complication [18-20], as well as its high in-hospital mortality rate [15], it is crucial to understand the association between COVID-19 and CVT, the clinical picture, and the treatment outcomes better. Here, we report a case of asymptomatic COVID-19 with massive CVT.

## Case presentation

A male patient in his 30s, with no known comorbidities, arrived at the emergency department complaining of severe headache and subjective weakness in the left arm. The headache was described as severe, generalized, and throbbing. It persisted for four days and was initially only associated with vomiting. The patient also expressed a weakness in his left arm that started 24 hours before his presentation. COVID-19 PCR swab was done as a part of hospital policy for any patient who requires admission to the hospital and resulted positive. He had no recent COVID-19 test done.

On systemic review, the patient denied any symptoms commonly reported in patients with COVID-19; he had no history of fever, coughing, chest pain, or difficulty in breathing. There were no reports of a witnessed seizure, loss of consciousness, or abnormal behavior. The patient had no diarrhea, abdominal pain, or burning micturition. He had no rash as well. He had no recent history of trauma. The patient reported that he neither drank alcohol nor smoked any sort of tobacco. The patient had no known family history of any blood disorders, strokes, or similar presentations. He had no recent travel history and no recent contact with any COVID-19-positive patient. He lived with a roommate, who was healthy and had no recent upper respiratory tract infection. The patient was unvaccinated, as the vaccination campaign was not yet started. 

On examination, his vital signs were stable. His blood pressure was 128/84 mmHg, pulse 100 beats per minute, respiratory rate 18 breaths per minute, oxygen saturation (SpO2) 100% on room air, and a temperature of 36.3 °C. On general examination, the patient had jerky movements in the left arm, but he was conscious and oriented with a Glasgow coma scale (GCS) of 15/15. He had no neck stiffness. The cranial nerve examination was normal. In the motor examination, his power in bilateral upper limbs and lower limbs was normal scoring 5/5 as there was an active movement of all limbs against gravity with full resistance. The cerebellar examination was normal. 

After the initial examination, considering the clinical presentation of the patient, he was sent for a brain CT scan; however, on the way to the radiology suite, he developed a tonic-clonic seizure. The emergency team intervened with 10 mg of diazepam. Nevertheless, the patient soon had recurrent episodes of seizure within 30 minutes and received 10 mg of diazepam; he was also loaded with 1 gram of levetiracetam, which aborted the seizures. The patient had a normal glucose level, with no hypoglycemia. Soon after, the patient had a third attack of seizure. The patient did not regain consciousness, and he was not able to maintain his airway. The patient’s GCS dropped to 8/15 with eye-opening 2/4, verbal response 2/5, and motor response 4/6. As a result, the emergency team decided to intubate him with rapid sequence intubation. Medications used for intubation were midazolam 20 mg as a pretreatment medication, propofol 100 mg and fentanyl 100 microgram were used for induction, and suxamethonium chloride 100 mg as a neuromuscular blocking agent. The readings of venous blood gas post-intubation is shown in Table 1:

**Table 1:** Venous blood gas post-intubation PCO2: partial pressure of carbon dioxide; HCO3: bicarbonate

Venous blood gas post-intubation	
pH	7.16 (low)
pCO2	48.6 mmHg (normal)
HCO3	15 mmol/L (low)
Lactic acid	14.5 (high)

**Figure 1:**
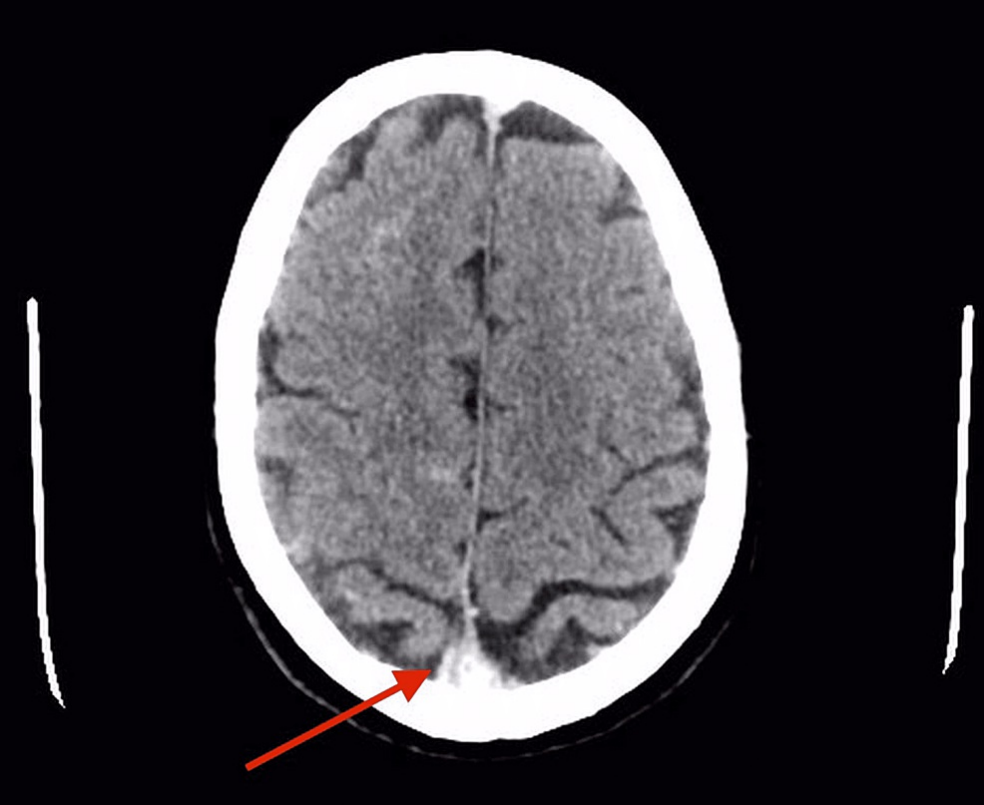
CT plain of the brain (axial section). The red arrow is pointing toward the superior sagittal sinus, which is hyperdense (positive delta sign), indicating there is a thrombus.

A chest x-ray was done and reported normal with an endotracheal tube in place. The CT brain and venogram scans were done after the patient was stabilized. The CT brain scan showed hyperdense superior sagittal sinus as shown in Figure 1, and superficial cortical veins bilaterally, which strongly suggested that the patient was having thrombosis. 

**Figure 2:**
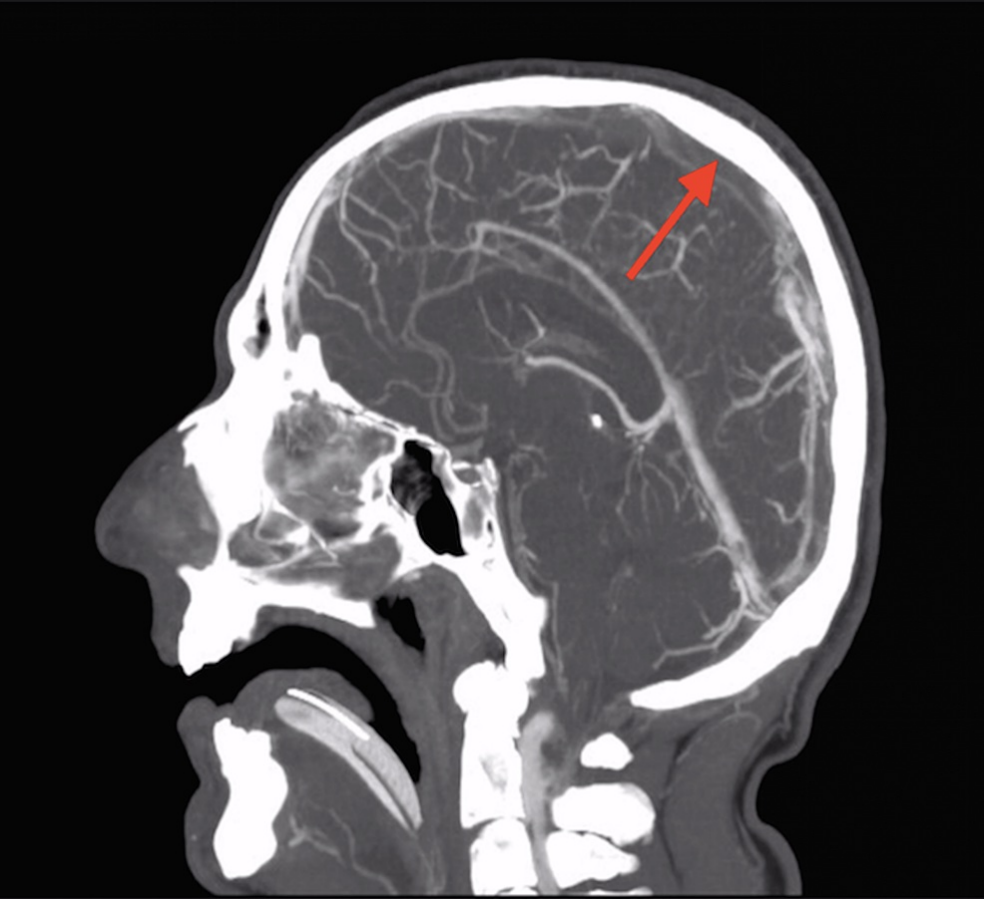
CT venogram of the brain (sagittal section). The red arrow is pointing toward the superior sagittal sinus where there is a massive thrombus (no contrast filling)

**Figure 3:**
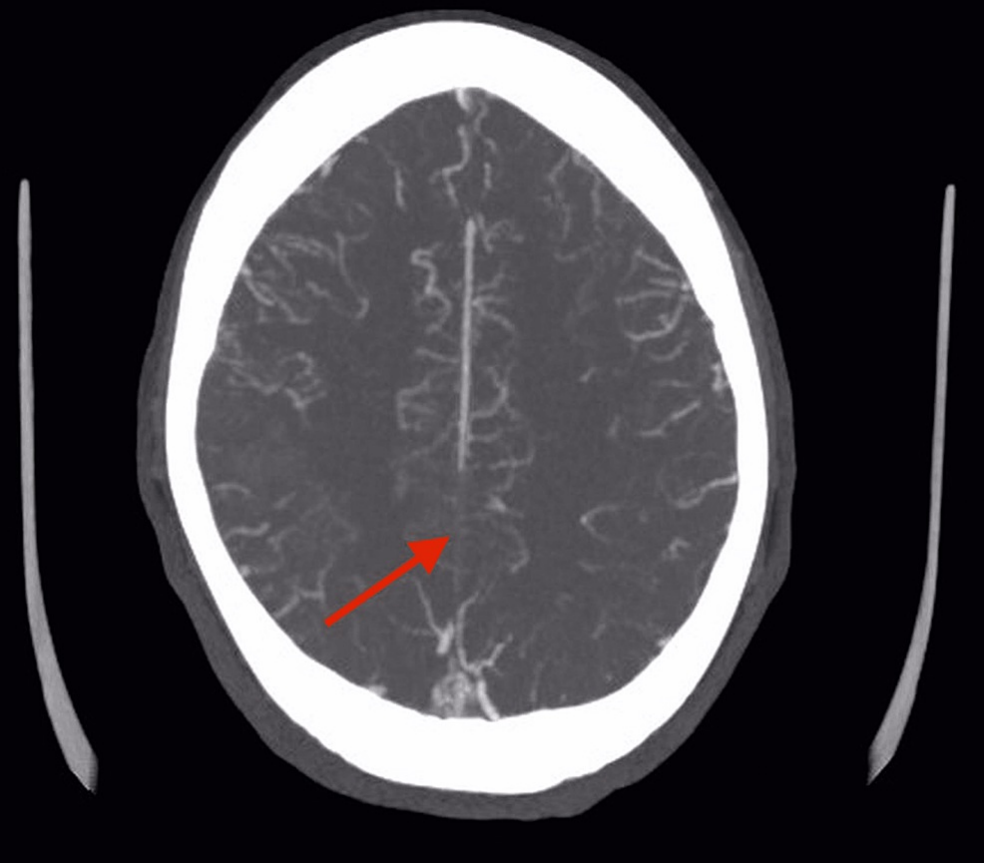
CT venogram of the brain (axial section). The red arrow is pointing toward the superior sagittal sinus where there is no contrast filling indicating a thrombus.

CT venogram done showed extensive thrombosis of the superior sagittal sinus and the superficial cortical veins bilaterally as shown in Figure 2 and Figure 3.

Apart from brain imaging, the initial blood investigations were performed on the same day of the patient presentation and are given in Table 2.

**Table 2:** Initial blood investigations results APTT: activated partial thromboplastin clotting time; INR: (international normalized ratio; LDH: lactate dehydrogenase

Blood tests results		
Full blood count	WBC count	11.9 10^3/uL (high)
	Neutrophil absolute	9.9 10^3/uL (high)
	Lymphocyte absolute	0.910^3/uL (low)
	Monocyte absolute	1.1 10^3/uL (high)
Inflammatory markers	Procalcitonin	0.06 ng/ml (high)
	C-reactive protein	10.7 mg/dl (high)
Coagulation profile	Prothrombin time	14.8 sec (high)
	APTT	34.4 secs (normal range 28-41 secs)
	INR	1.15 (high)
	D-dimer	4.90 ug/ml (high)
	Protein C&S values	normal
	Thrombophilia factor II and Factor V Leiden test	normal
	Fibrinogen	334 mg/dL (normal)
Other blood tests	LDH	180 U/L (normal)
	Ferritin	131.0 ng/ml (normal)

Initially, differentials to the patient's clinical presentation were limited to investigating the presenting focal seizures; a history of alcohol consumption was ruled out, and a plan to rule out traumatic cerebral injury, hypoperfusion, or hemorrhage was made. Parasitic infections such as neurocysticercosis or malaria may also present similarly, but those were also ruled out by a CT brain. Brain imaging revealed evidence of CVT. With the current reality of the COVID-19 pandemic, it was critical to consider this virus as a cause of the patient's hypercoagulable state and thrombosis. 

Further investigations were done as an inpatient to rule out other diseases. Differential diagnoses that were sought included relevant clotting factor disorders such as protein C and S deficiencies, thrombophilia, including mutations in Factors II and Factor V Leiden and labs reported negative. A lupus anticoagulant profile was done including anticardiolipin IgM/IgG and antinuclear antibodies (ANA), which were reported negative. An extractable nuclear antigen (ENA) panel was done to rule out autoimmune diseases and resulted negative. A thyroid function test was done and the results were within normal ranges. Urine culture, blood culture, and respiratory culture were all performed on admission, and the final result showed no growth.

In relation to the management, in the emergency department, the patient received diazepam and levetiracetam after developing multiple seizure attacks. Moreover, he was intubated and sedated because of the low GCS and unsecured airway. As meningitis was one of the differentials, ceftriaxone, acyclovir, and dexamethasone were administered.

**Figure 4:**
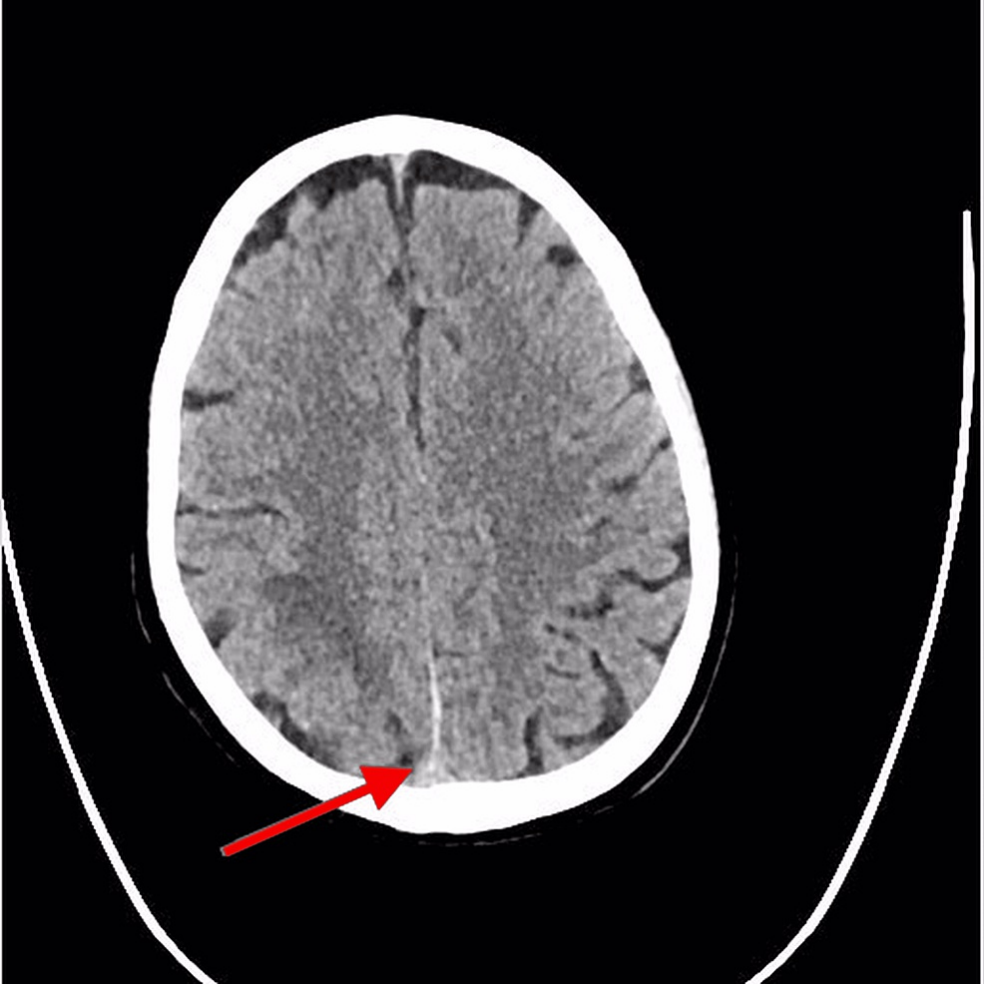
Follow-up plain CT brain (axial section), red arrow pointing towards the superior sagittal sinus showing resolution of the hyperdensity.

The patient was admitted to the ICU. He was intubated and sedated. The patient was continuously monitored with frequent arterial blood gas tests and had an arterial line placed for blood pressure monitoring. On day three of admission, the patient had no seizures and was planned to wean off sedation. After the patient was extubated, he was shifted to a regular isolation ward. A follow-up CT brain was done after 10 days of admission and it was seen that the previously thrombosed cerebral venous sinuses and cortical veins were now less hyperdense, suggesting recanalization, which is a favorable regressive course. There was resolution of the hyperdensity in the superior sagittal sinus (no delta sign) on the plain CT brain as shown in Figure 4.

Throughout the admission, he was kept on enoxaparin injections and then started on warfarin. The patient's (international normalized ratio (INR) was checked daily until a target INR of 2.93 was reached. The patient was not started on any medication for the COVID-19 virus and was given supportive care. As per the hospital protocol, the patient was kept in isolation and shifted out of isolation after completing 14 days of isolation and receiving two negative COVID-19 swab tests. He remained asymptomatic throughout the admission and did not suffer from any further neurologic deficits. He was discharged home on warfarin 6 mg once per day and levetiracetam 1500 mg twice per day. Also, he was given a follow-up appointment with the neurology clinic. Despite the scheduled follow-up appointment, the patient preferred to go back to his home country and follow up.

## Discussion

We describe a case of CVT in a patient with respiratory asymptomatic COVID-19 infection. Our patient had no known risk factors for thrombosis. This case highlights the importance of early recognition of possible thrombosis as a complication in patients with COVID-19 and high D-dimer levels regardless of the clinical presentation.

CVT

Manifestations of CVT

The most common manifestation of CVT is headache with a variable degree of pain in 80-90% of patients [1]. Other manifestations of CVT include vomiting, seizure, and altered mental status. A motor deficit, sometimes with seizures, can also occur. In addition, scalp edema and dilated scalp veins may be seen on examination [2].

Predisposing Factors to CVT

 There are multiple predisposing causes of CVT. The risk factors for venous thrombosis, in general, are linked classically to the Virchow triad of stasis of the blood, changes in the vessel wall, and changes in the composition of the blood. Risk factors are divided into acquired risks (e.g., surgery, trauma, pregnancy, puerperium, antiphospholipid syndrome, cancer, exogenous hormones and infections) and genetic risks (inherited thrombophilia). Saposnik et al. classified the risk factors prevalences, and the highest in the list was oral contraceptive pills with a prevalence of 54.3%, prothrombotic conditions (34.1%), and pregnancy and puerperium (21%), followed by infections including parameningeal infections accounting for 12.3% [3].

COVID-19

COVID-19 is commonly known to affect the respiratory system. Neurological symptoms due to thromboembolic events were also reported in many cases. For instance, in 2020, a retrospective observational study done on 214 cases in three different hospitals in China showed various neurological manifestations of COVID-19 disease; among those presentations, only two patients had a seizure [4]. Furthermore, other published studies show the occurrence of venous thromboembolism and stroke in approximately 20% and 3% of patients, respectively [5].

COVID-19 and Hypercoagulable State Mechanism

COVID-19 leads to a hypercoagulable state in which the mechanism is not well known yet. One of the proposed mechanisms states that cytokine storm in COVID-19 patients leads to the pro-coagulable state [6]. Another mechanism asserts that the COVID-19 virus is procoagulant on its own as it has angiotensin-converting enzyme-2 (ACE-2), receptors. These receptors are suggested to play a role in viral adherence to cells leading to inflammatory cell infiltrations and prothrombotic events [7].

The incidence of venous thromboembolism is high in COVID-19 infection. Jimenez did a meta-analysis based on the type of venous thromboembolism, including deep venous thrombosis (DVT) and pulmonary embolism, which showed that DVT incidence was 12.1%. In comparison, the incidence of pulmonary embolism was 7.1% [8].

It is proposed that the level of D-dimer is proportional to the severity of the COVID-19 infection [9]. A literature review by Al-Ani reported that one of the earliest and most common laboratory findings noted in COVID-19 patients requiring hospitalization was the marked elevation of D-dimer. A high D-dimer is nonspecific and is often associated with various medical conditions such as infections, trauma, or hospitalization. However, in the setting of COVID-19 disease, it has been consistently reported as a prognostic marker that is associated with a critical course and higher mortality [10]. A study conducted by Tang on 183 COVID-19 patients revealed that the lab values of D- dimer is up to four-fold higher in COVID patients. The D-dimer level was done upon admitting the patients, and they noted that in COVID survivors, the level ranged between 0.35-1.29 μg/mL, but the non-survivors ranged between 0.77-5.27 μg/mL, with the normal value <0.5 μg/mL [11]. A retrospective evaluation by Guan et al. for 1099 patients with COVID-19 infection suggests that a cut-off point of 0.5 mg/L is more frequent in patients with severe disease than those without it [12].

A cohort study conducted on 24 COVID-19 patients admitted to the ICU; suggests that patients with COVID-19 may develop a state of hypercoagulability as evident by increased factor VIII, von Willebrand factor, and fibrinogen levels. This hypercoagulability could contribute (in addition to other causes) to the development of pulmonary embolism and/or deep vein thrombosis of the lower limbs. However, the reasons for the observed hypercoagulability are unknown. Plasma hypercoagulability may occur due to increased procoagulant factors, decreased levels of anticoagulant factors, or both. In this cohort, factor VIII, one of the most potent triggers of hypercoagulability, is strongly increased (up to 460 U/dL), and the main anticoagulants are (near)normal (i.e., antithrombin) or even increased (i.e., protein C) [13].

Sarkar et al. conducted a literature review about COVID-19 and coagulopathy in which they compared the features of disseminated intravascular coagulation and COVID-19 coagulopathy. In COVID-19, the levels of D-dimer are higher than the D-dimer level in other infections. The prothrombin time and activated partial thromboplastin clotting Time (aPTT) are normal or mildly raised fibrinogen level is raised in contrast to sepsis-induced disseminated intravascular coagulation (DIC). These factors all contribute to the fact that thrombosis is more common than bleeding in COVID-19 infection [14].

The link between CVT and COVID-19 infection

COVID-19 has been associated with ischemic strokes, seizures, and encephalopathy but there are very few reports of COVID-19 related to CVT in the published literature. A meta-analysis conducted by Baldini reveals that the frequency of CVT among hospitalized COVID-19 patients is 0.08%. They had 57 cases of CVT and 10 cases were presented with either focal or generalized tonic-clonic seizures. The remaining 47 cases had CT-angiography done, and this showed that 29 cases had multiple CVT. Most patients had thrombosis in the transverse sinus, followed by the sigmoid sinus and the superior sagittal sinus [15]. Case reports were published reporting the manifestations of CVT in COVID-19 patients. Two case reports discussed that isolated CVT could occur in young and healthy patients with no risk factors [16,17] as in our case, in which the patient had no known comorbidities or risk factors. A case report showed that thrombotic events could be a manifestation of a COVID-19 infection [18]. Similarly, as with our case, the patient had no respiratory symptoms of COVID-19 disease and remained asymptomatic throughout the whole admission. However, multiple case reports were published concerning COVID-19 and CVT, but in those cases, the patients had symptoms of COVID-19 infection [16-18]. Another case was reported by Kaur and discussed a case of a CVT patient with no risk factors but positive COVID-19 IgG only. They concluded that COVID-19 was the cause of CVT after ruling out all of the risk factors [19].

To further elaborate on the relationship between COVID-19 symptoms and the neurological symptoms of CVT, studies showed that there is no relation between the onset of systemic COVID-19 symptoms and CVT diagnosis. The time between the onset of infection to the neurological symptoms of CVT can range from the same day up to four months [15,20]. 

## Conclusions

COVID-19 infection does not always manifest with respiratory tract infection symptoms; patients can have neurological symptoms. Further investigation is required when we suspect thrombotic events in COVID-19 patients to initiate early therapy in such patients. 
